# A stacking ensemble machine learning model to predict alpha-1 antitrypsin deficiency-associated liver disease clinical outcomes based on UK Biobank data

**DOI:** 10.1038/s41598-022-21389-9

**Published:** 2022-10-11

**Authors:** Linxi Meng, Will Treem, Graham A. Heap, Jingjing Chen

**Affiliations:** 1 Florida State University, Tallahassee, USA; 2 Takeda Development Center Americas, Inc., Cambridge, MA USA

**Keywords:** Diseases, Medical research, Drug development

## Abstract

Alpha-1 antitrypsin deficiency associated liver disease (AATD-LD) is a rare genetic disorder and not well-recognized. Predicting the clinical outcomes of AATD-LD and defining patients more likely to progress to advanced liver disease are crucial for better understanding AATD-LD progression and promoting timely medical intervention. We aimed to develop a tailored machine learning (ML) model to predict the disease progression of AATD-LD. This analysis was conducted through a stacking ensemble learning model by combining five different ML algorithms with 58 predictor variables using nested five-fold cross-validation with repetitions based on the UK Biobank data. Performance of the model was assessed through prediction accuracy, area under the receiver operating characteristic (AUROC), and area under the precision-recall curve (AUPRC). The importance of predictor contributions was evaluated through a feature importance permutation method. The proposed stacking ensemble ML model showed clinically meaningful accuracy and appeared superior to any single ML algorithms in the ensemble, e.g., the AUROC for AATD-LD was 68.1%, 75.9%, 91.2%, and 67.7% for all-cause mortality, liver-related death, liver transplant, and all-cause mortality or liver transplant, respectively. This work supports the use of ML to address the unanswered clinical questions with clinically meaningful accuracy using real-world data.

## Introduction

Alpha-1-antitrypsin deficiency (AATD) is an autosomal codominant genetic disorder with a prevalence range of 1 per 2500 to 1 per 5000 individuals in Europe and North America that causes early pulmonary disease in adults and liver disease in children and adults^1^, and which often goes underdiagnosed. Alpha-1-antitrypsin (AAT), also known as *SERPINA1* (serine protease inhibitor, group A, member 1), is a 52 kDa circulating glycoprotein protease inhibitor of the serpin family. Its primary function is to inhibit neutrophil elastase and other proteases to prevent excessive protease-induced tissue damage^2,3^. AAT is normally synthesized primarily in hepatocytes and secreted in monomeric form. If the AAT proteins are malformed or deficient, it may lead to predisposition for obstructive pulmonary disease and/or liver disease^1^. The PiZZ genotype is known as the most common deficiency genotype and tends to result in the worst clinical presentation^4^. Data from real-world clinical practice has shown that over 90% of AATD is due to the PiZZ genotype^5^. The milder genotypes such as PiSZ and PiMZ are also linked to the development of lung and liver disease, mainly when unhealthy behaviors such as smoking or alcohol use are present^4^. Clinical research shows approximately 40% of adult AATD patients dying of all causes had cirrhosis at the time of death^6^, and approximately 15% of adult patients with AATD-associated liver disease (AATD-LD) required liver transplantation^7^. Although clinical trials are underway, there is no approved therapy for AATD-LD. Liver transplantation is the only curative treatment available so far.

### Motivations

The signs of AATD-LD include elevated transaminases or bilirubin, hepatitis, hepatic fibrosis or cirrhosis^8^. It is known that liver damage may progress slowly for decades before clinical presentation. Disease progression can be accelerated significantly by other factors, including nonalcoholic fatty liver disease, alcoholic liver disease, hepatitis, alcohol consumption, smoking, etc. These factors can also cloud accurate diagnosis of AATD-LD^9–11^. Thus, predicting the clinical outcomes of AATD-LD and defining patients who are more likely to progress to advanced liver disease is crucial to enable timely medical intervention. It will also enable researchers to make data-driven decisions to inform clinical outcome endpoint selection and clinical development strategy when designing clinical trials for potential AATD-LD therapies. However, the risk factors potentially contributing to the progression of AATD-LD have been poorly studied^12^. The existing clinical research on AATD-LD mainly utilizes conventional correlation analysis or multivariate regression analysis based on a limited number of predictor variables such as demographics, baseline disease characteristics, serum tests and lifestyle^7,13–20^. Many studies have limitations due to small sample size or older data^7^. None of the studies has compared the risk factors between AATD-LD and other liver disease. Although machine learning (ML) techniques have been applied in the medical field for disease diagnosis and treatment outcome prediction, given recent advances in ML algorithms and statistical computing power^21–31^, little research has been done to understand the AATD-LD patients’ journey or predict the disease progression of AATD-LD using ML algorithms.

### Objectives

We were intrigued and aimed to fill the gaps by applying advanced ML to predict the disease progression of AATD-LD using real-world data from the UK Biobank. In this work, we aimed to:

- establish a predictive ML model of clinical outcomes to assess disease progression of AATD-LD based on generally available clinical information collected in daily practice;
- improve the ML model prediction by applying a supervised stacking ensemble learning technique by combining multiple ML algorithms including random forest (RF), elastic net regularized regression (ENRR), gradient boosting (GB), and artificial neutral network multilayer perceptron (ANN-MLP) via meta-learning; and
- improve the interpretability of predictive ML model by mapping the importance of predictor contributions through a feature importance permutation method.

This article is organized as follows: the basic concepts of the supervised stacking ensemble learning technique, a brief overview of data and analysis pipeline, and the ML model training and testing workflow are described in the “Methods” section. We present the proposed predictive ML model for AATD-LD based on real-world data from the UK Biobank as well as the model performance evaluation and model interpretation in the “Results” section. A brief discussion on the impact of this work is provided in the “Discussion” section. Of note, we trained the ML model for AATD-LD and any liver disease for comparative purpose. Our work focused on the prediction of disease progression of AATD-LD, but it can be applied to other clinical outcomes and/or diseases. In summary, the generalizable predictive patterns revealed in this work support the potential of ML model as a new tool to address the unanswered clinical questions with clinically meaningful accuracy using real-world data.

## Methods

This section provides a brief overview of data and analysis pipeline, data assembly and process prior to the modeling training, and the ML model building workflow in this work. We also provide the details of statistical techniques applied to improve the model performance and interpretation including feature selection, oversampling technique and feature importance. The principles that we demonstrated in this work can be readily applied to other clinical outcomes and/or disease indications. This study (UK Biobank application #26041) was covered by the general ethical approval for UK Biobank studies. As per informed consent procedures, informed consent was obtained and all participant data was anonymized. All methods were carried out in accordance with relevant guidelines and regulations.

### Data and patient selection

Patient data were extracted from the UK Biobank (https://biobank.ctsu.ox.ac.uk), a large-scale biomedical database, of which 500,000 patients aged 40 to 69 years recruited throughout the UK between 2006 and 2010. The database included patients with a wide range of serious and life-threatening illnesses. Patients had undergone measures, provided blood, urine and saliva samples, and detailed information about themselves, and agreed to have their health followed^32,33^. The blood, urine and saliva samples were stored in such a way as to allow different types of assay to be performed (e.g., genetic, proteomic and metabonomic analyses)^33^. Demographic and behavioral information was recorded using self-reported questionnaires during clinic visits. The UK Biobank data included lifestyle, medical history and sociodemographics, physical and environmental measures (including urinary biomarkers, cognitive function and hearing tests), genetic data and health outcome data^34^.

All data used in this work were extracted from UK Biobank (application #26041) for 11,583 patients with a diagnosis of any liver disease according to International Classification of Diseases (ICD) codes. Four hundred and fifty-five patients with a diagnosis of AATD-LD (identified by ICD code), including 20 AATD-LD patients with the PiZZ genotype (SNP rs28929474), were subsequently identified. The demographic and disease characteristics for the patients of interest are shown in Table 1.

**Table 1:** Summary of demographic and disease characteristics in patients with any liver disease and AATD-LD.

Variables	Category	Any liver disease (N = 11,583)	AATD-LD (N = 455)
Sex, n (%)	Female	6097 (52.6%)	226 (49.7%)
	Male	5486 (47.4%)	229 (50.3%)
	Missing	0	0
Race, n (%)	White	10,840 (94.1%)	426 (94.2%)
	Non-white	674 (5.9%)	26 (5.8%)
	Missing	69	3
Obesity, n (%)	Non-obese	7180 (62.7%)	321 (72.0%)
	Obese	4278 (37.3%)	125 (28.0%)
	Missing	125	9
Diabetes, n (%)	Non-diabetic	9862 (85.9%)	401 (88.9%)
	Diabetic	1625 (14.1%)	50 (11.1%)
	Missing	96	4
Smoking status, n (%)	Never smoking	5090 (44.3%)	182 (40.3%)
	Past smoker	4493 (39.1%)	194 (42.9%)
	Current smoker	1911 (16.6%)	76 (16.8%)
	Missing	89	3
Age (years)	Mean	58.5	60.3
	Min, max	40, 70	41, 70
	Missing	0	0
BMI (kg/m^2^)	Mean	29.0	27.5
	Min, max	15.0, 69.0	16.9, 52.5
	Missing	125	9
Weight (kg)	Mean	82.0	78.0
	Min, max	35.8, 190.0	41.7, 151.4
	Missing	122	7
Waist (cm)	Mean	95.4	92.9
	Min, max	57, 171	62, 153
	Missing	84	4

Patients with any liver disease were identified by ICD code. Patients with AATD-LD is a subset of patients with any liver disease.

### Clinical outcomes

The clinical outcomes of interest to assess the disease progression of AATD-LD and any liver disease included:

1. all-cause mortality (taken from UK Biobank—death register),
2. liver-related death (a subset of all-cause mortality with liver disease diagnosis),
3. liver transplant (taken from UK Biobank—summary of operations and identified by OPCS4 code), and
4. all-cause mortality or liver transplant, a combination of clinical outcomes (1), (2), and (3).

The frequency of these outcomes recorded among study patients is shown in Table 2.

**Table 2:** Summary of clinical outcomes in patients with any liver disease and AATD-LD.

Clinical outcomes, n (%)	Any liver disease (N = 11,583)	AATD-LD (N = 455)
All-cause mortality	3524 (30%)	245 (54%)
Liver-related death	1230 (10%)	41 (9%)
Liver transplant	124 (1%)	5 (1%)
All-cause mortality or liver transplant	3619 (31%)	246 (54%)

### Predictors and feature selection

All potential predictor variables collected in the UK Biobank were included in the analysis and categorized into four predictor blocks to facilitate interpretation of the prediction results, as shown in Table 3.

- Predictor Block 1: baseline demographics;
- Predictor Block 2: baseline disease characteristics;
- Predictor Block 3: lifestyle and others; and
- Predictor Block 4: baseline laboratory parameters.

**Table 3:** Description of potential predictor variables.

Category	Description
**Predictor Block 1** Demographics	Age Age of diagnosis Gender Ethnicity BMI Weight Waist circumference
**Predictor Block 2** Baseline disease characteristics	Other underlying conditions Non-alcoholic steatohepatitis Lung disease Diabetes Obesity
**Predictor Block 3** Lifestyle and others	Alcohol intake/status Smoking status Medical procedure Major operation
**Predictor Block 4** Baseline laboratory parameters	Blood assays Albumin Alanine aminotransferase Aspartate aminotransferase Alkaline phosphatase Gamma-glutamyl transferase (GGT) Total bilirubin Direct bilirubin International normalised ratio Hemoglobin A1c Total protein Spirometry Forced vital capacity (FVC) Forced expiratory volume in 1 s (FEV1) Peak expiratory flow (PEF)

Variables in predictor blocks 2 and 3 were obtained from patient-reported questionnaires. There may be more than one predictor variable in each predictor category. 58 predictor variables were identified via feature selection prior to ML model training.

**Figure 1:**
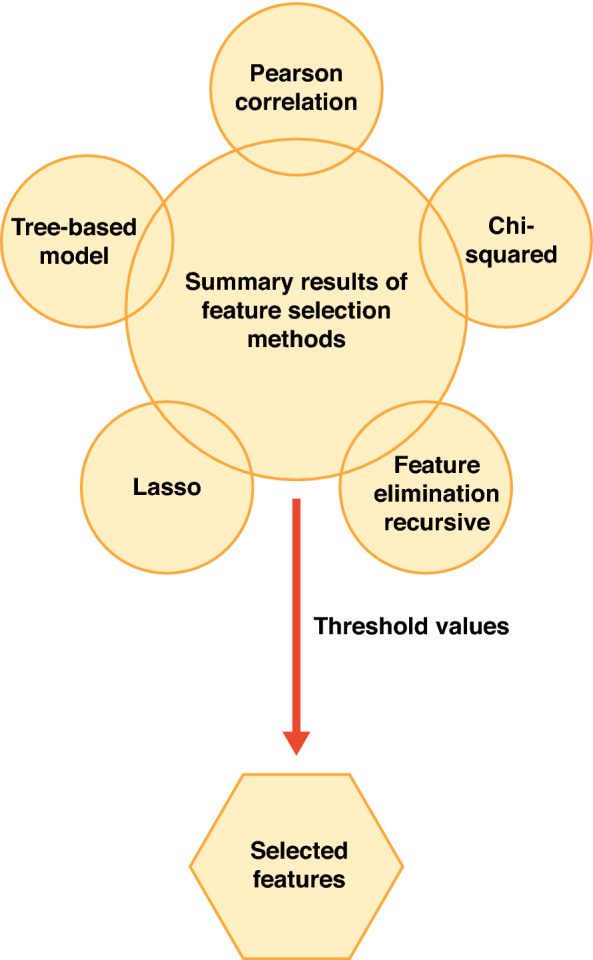
Feature selection strategy prior to model training.

Of note, there are multiple variables with similar information in each predictor block. To prevent the modeling barriers from the overfitting or multicollinearity, redundant features were eliminated through feature selection methods prior to the model training. The final set of predictor variables for the model training was selected through the joint application of seven feature selection methods including: (1) filter methods, such as Pearson correlation and Chi-squared correlation; (2) wrapper methods, such as feature elimination recursive; and (3) embedded methods such as Lasso and three tree-based models, as shown in Fig. 1. Predictor variables selected by at least 4 of the 7 feature selection methods were identified as the final potential predictors for the clinical outcomes and included in the ML model training. There were 58 predictor variables in total identified through the feature selection process.

### Data assembly and analysis pipeline

**Figure 2:**
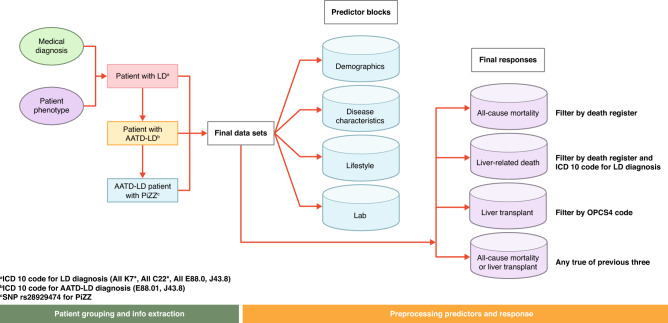
Flow chart of data assembly, processing, and analysis.

The data were preprocessed before modeling (e.g., centering and scaling the predictors, imputing the missing predictor information via multiple imputation). The process flow for data assembly, processing, and analysis is shown in Fig. 2. Complete algorithms of data domains and data classifiers used in this work can be found in Supplementary Appendix C.

### Oversampling technique

To address the imbalanced classification challenge where there were too few records of a minority class for the model to effectively learn and to improve the model performance on the minority class, the synthetic minority oversampling technique (SMOTE)^35^ was applied to the clinical outcomes with data imbalance including liver-related death and liver transplant. The new synthetic records were generated using the existing samples of the minority class by linear interpolating for the minority class. AUPRC was used as a performance measure for data imbalance.

### Stacking ensemble algorithm

In the practice of ML, the choice of ML model is critical to obtain good results. The real challenge is to explore the space of possible ML models and identify a robust model with good prediction performance. As an older saying says “unity is strength”, we aimed to apply an ensemble method based on a hypothesis that combination of multiple ML models will produce a more powerful and robust model. Stacking is one of the ensemble methods and meta-learning algorithms that minimizes the variance, reduces the bias and improves the model predictive force by combing multiple heterogeneous base ML models into one meta-model to output the predictions based on the multiple predictions from the base ML models. The final meta-model can be viewed as a correction of base models or a weighted average of base models.

Therefore, the stacking ensemble learning algorithm^36,37^ was applied in this work in order to achieve an optimal model prediction performance. The stacking ensemble is a meta-learning algorithm that combines the predictions from multiple well-performing ML models including classification tree and/or regression methods to make the final model perform better than any single model in the ensemble. We applied and combined the learning from random forest (RF), gradient boosting (GB), elastic net regularized regression (ENRR), and artificial neural networks-multilayer perceptron (ANN-MLP) into the stacking ensemble learning algorithm. The technical details of the stacking learning algorithm^37^ are presented in Supplementary Appendix A.

- *RF* is an ML algorithm for classification, which consists of a large number of individual decision trees, and uses bagging and feature randomness for training to create an uncorrelated forest of trees. The final prediction from random forest model is the class selected by most trees.
- *GB* is an ML algorithm that uses boosting technique and grows trees in a stage-wise, gradual, additive and sequential manner. Two GB algorithms were applied in this work, including eXtreme Gradient Boosting (XGBOOST), which splits the tree level-wise and light GBM, which has faster training speed and higher efficiency.
- *ENRR* is an application of regularized regression with penalties to avoid extreme parameters that could cause overfitting. ENRR combines two commonly used regularization techniques (Lasso and Ridge) into a hybrid penalized model.
- *ANN* is one of the deep-learning algorithms inspired by the structure and function of the human brain. MLP is a class of feedforward ANN. We applied multiple-input single-output neural network forecasting in this work.

### Nested five-fold cross-validation

To optimize the stability of the prediction results, a nested five-fold cross-validation with independent random partitions was conducted with 100 repetitions. The nested cross-validation has an inner loop cross-validation nested in an outer loop cross-validation, where the inner loop was used for model selection and hyperparameter tuning and the outer loop was used for model performance evaluation. The full dataset was split into the *Training Set* and *Test Set* prior to the model building through the nested five-fold cross-validation method. The *Training Set* was implemented to build up and train the model, and the *Test Set* was used to validate the model built. Of note, the SMOTE oversampling technique was applied to the *Training Set* for liver-related death and liver transplant. To avoid the noisy estimate of model performance by a single run of nested five-fold cross-validation, we conducted different splits of *Training* and *Test* data by repeating the nested five-fold cross-validation 100 times to stabilize the performance of the ML models.

### Model performance evaluation metrics

The model performance was evaluated by prediction accuracy, AUROC, and AUPRC. The prediction accuracy and the AUROC were reported as a performance measure to indicate the capability of a classification model to distinguish between classes. A prediction accuracy or AUROC score close to 1 indicated good model separability. The AUPRC was reported as a performance metric for imbalance data. An AUPRC score better than the baseline fraction of positive cases indicated good performance. The mean result and standard deviation across all iterations were reported. It is worth pointing out that the mean result is considered as a more accurate and stable estimate to the underlying performance of model prediction.

**Figure 3:**
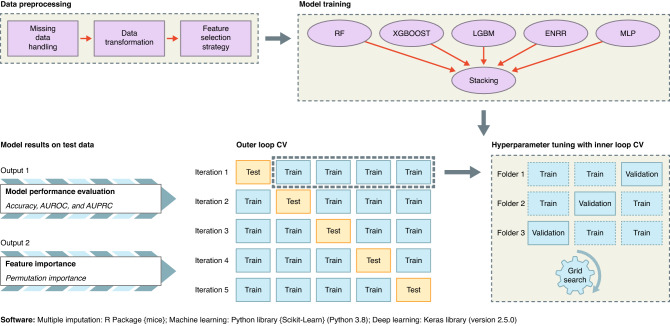
The workflow of stacking ensemble learning.

This analysis was carried out using Python 3.8 and Keras 2.5.0. Figure 3 presents the workflow of the stacking ensemble learning algorithm in this work.

### Feature importance

Feature importance refers to a class of techniques for assigning scores to input features in a predictive model that indicates the relative importance of each feature when making a prediction, providing insight and better understanding into the data and an ML prediction model. We applied the permutation importance^38^ to each of the five ML models to obtain the permutation importance scores and calculated the final feature importance score by summing up these importance scores (Supplementary Appendix B). The important predictors were identified and ranked based on the final importance score.

## Results

### Predictive performance of stacking ensemble model

Table 4 displays the model performance measures (prediction accuracy, AUROC, and AURPC) using the stacking ensemble learning algorithm in the *Training Set* and *Test Set* for the four clinical outcomes of interest in patients with AATD-LD, while Table 5 displays the model performance measures in patients with any liver disease for comparative purpose. The results show that the stacking ensemble model performed similarly in patients with AATD-LD versus patients with any liver disease, with acceptable and clinically meaningful accuracy in both the *Training Set* and the *Test Set*. The stacking ensemble models worked particularly well for predicting liver-related death and liver transplant.

**Figure 4:**
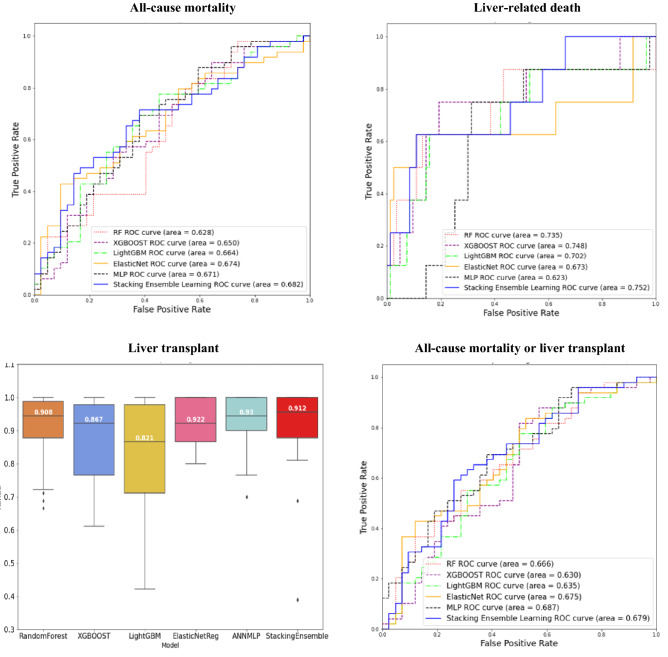
ROC curves for the trained classifiers in the *Test Set* from one *Training-Set* split for patients AATD-LD (N = 445). Given the low liver transplant event incidence in AATD-LD patients in the *Test Set*, there was insufficient data to populate the ROC curve for liver transplant and a box plot was presented instead.

**Figure 5:**
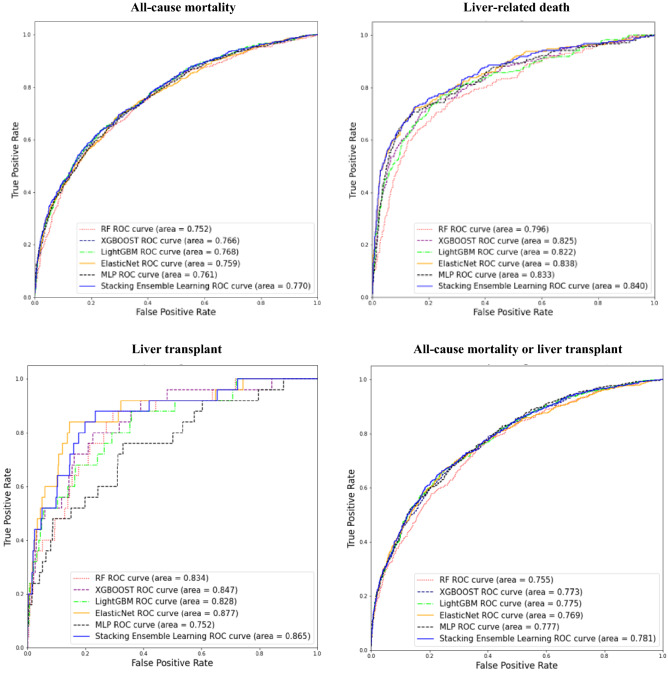
ROC curves for the trained classifiers in the *Test Set* from one *Training-Set* split for patients with any liver disease (N = 11,583).

- *AATD-LD* The mean prediction accuracy was 0.828 and 0.632 for all-cause mortality, 0.991 and 0.914 for liver-related death, 1.000 and 0.989 for liver transplant, and 0.837 and 0.633 for all-cause mortality or liver transplant in the *Training Set* and *Test Set* of patients with AATD-LD, respectively (Table 4). The mean AUROC was 0.899 and 0.681 for all-cause mortality, 0.997 and 0.759 for liver-related death, 1.000 and 0.912 for liver transplant, and 0.903 and 0.677 for all-cause mortality or liver transplant in the *Training Set* and *Test Set* respectively. For illustration purposes, Fig. 4 displays the receiver operating characteristic (ROC) curve of the final best stacking ensemble model compared with each of the five base ML models in the *Test Set* from one *Training-Test* split of AATD-LD*.*
- *Any liver disease* The mean prediction accuracy was 0.806 and 0.756 for all-cause mortality, 0.911 and 0.913 for liver-related death, and 0.999 and 0.989 for liver transplant, and 0.815 and 0.755 for all-cause mortality or liver transplant in *Training Set* and *Test Set* of patients with any liver disease, respectively (Table 5). The mean AUROC was 0.852 and 0.770 for all-cause mortality, 0.999 and 0.835 for liver-related death, 1.000 and 0.859 for liver transplant, and 0.863 and 0.777 for all-cause mortality or liver transplant in the *Training Set* and *Test Set*, respectively. Figure 5 displays the ROC of the final best stacking ensemble model in the *Test Set* from one *Training-Test* split of patients with any liver disease*.*

**Table 4:** Mean (± standard deviation) model performance measures for stacking ensemble learning in *Training Set* and *Test Set* across the nested five-fold cross-validation with 100 repetitions in patients with AATD-LD (N = 455), respectively.

Clinical outcomes	Accuracy		AUROC		AUPRC	
	Training	Test	Training	Test	Training	Test
All-cause mortality	0.828 ± 0.091	0.632 ± 0.038	0.899 ± 0.080	0.681 ± 0.035	0.911 ± 0.073	0.709 ± 0.032
Liver-related death	0.991 ± 0.011	0.914 ± 0.009	0.997 ± 0.007	0.759 ± 0.108	0.979 ± 0.043	0.411 ± 0.170
Liver transplant	1.000 ± 0.001	0.989 ± 0.000	1.000 ± 0.000	0.912 ± 0.133	1.000 ± 0.000	0.414 ± 0.416
All-cause mortality or liver transplant	0.837 ± 0.087	0.633 ± 0.029	0.903 ± 0.076	0.677 ± 0.025	0.917 ± 0.067	0.703 ± 0.040

*AUROC =* area under the receiver operating characteristic, *AUPRC =* area under the precision-recall curve.

**Table 5:** Mean (± standard deviation) model performance measures in *Training Set* and *Test Set* across the nested fivefold cross-validation with 100 repetitions in patients with any liver disease (N = 11,583).

Clinical outcomes	Accuracy		AUROC		AUPRC	
	Training	Test	Training	Test	Training	Test
All-cause mortality	0.806 ± 0.025	0.756 ± 0.008	0.852 ± 0.034	0.770 ± 0.009	0.737 ± 0.049	0.629 ± 0.016
Liver-related death	0.991 ± 0.011	0.913 ± 0.004	0.999 ± 0.002	0.835 ± 0.009	0.998 ± 0.003	0.517 ± 0.023
Liver transplant	0.999 ± 0.001	0.989 ± 0.003	1.000 ± 0.000	0.859 ± 0.045	1.000 ± 0.000	0.142 ± 0.048
All-cause mortality or liver transplant	0.815 ± 0.039	0.755 ± 0.006	0.863 ± 0.046	0.777 ± 0.010	0.764 ± 0.067	0.636 ± 0.010

*AUROC =* area under the receiver operating characteristic, *AUPRC* = area under the precision-recall curve.

### Overall predictive performance evaluation

We found the stacking ensemble model and five base ML models (including RF, XGBOOST, LGBM, ENRR, and ANN-MLP) used to train the stacking ensemble model all worked well with complex data and a massive scope of predictors, showing similar prediction performances. Tables 6 and 7 present the predictive performance evaluation metrics of the stacking ensemble model and each base ML model in patients with AATD-LD and patients with any liver disease, respectively. The results show that the stacking ensemble model achieved the best performance compared with each individual base ML model*,* yielding higher prediction accuracy and AUROC in both the *Training Set* and the *Test Set* for all four clinical outcomes. It is worth noting that each base ML model performed acceptably well but no one base ML model consistently outperformed others for all clinical outcomes. In summary, our results show that each base ML model improved in performance when combined with meta-learning, i.e., the proposed stacking ensemble learning predictive model.

- *AATD-LD* Among five base ML models, ENRR appeared to have the highest prediction accuracy for all-cause mortality (accuracy = 0.620 compared with 0.589, 0.614, 0.616, 0.595*)*; LGBM for liver-related death (accuracy = 0.897 compared with 0.894, 0.893, 0.851, 0.770); RF, LGBM, and ENRR for liver transplant (accuracy = 0.988 compared with 0.981, 0.982); and ENRR for all-cause mortality or liver transplant (accuracy = 0.641 compared with 0.620, 0.603, 0.605, 0.624) (Table 6).
- *Any liver disease* Among five base ML models, ENRR appeared to have the highest prediction accuracy for all-cause mortality (accuracy = 0.754 compared with 0.700, 0.724, 0.726, 0.746); LGBM for liver-related death (accuracy = 0.900 compared with 0.818, 0.897, 0.890, 0.878); LGBM for liver transplant (accuracy = 0.987 compared with 0.947, 0.985, 0.983, 0.979), and ENRR for all-cause mortality or liver transplant (accuracy = 0.753 compared with 0.707, 0.727, 0.728, 0.753, 0.740) (Table 7).

**Table 6:** Overall predictive model performance measures of stacking ensemble and each base model used in the ML training in patients with AATD-LD.

Model	Performance measure	All-cause mortality (N = 455)		Liver-related death (N = 455)		Liver transplant (N = 455)		All-cause mortality or liver transplant (N = 455)	
		Training	Test	Training	Test	Training	Test	Training	Test
**Stacking ensemble learning**	**Accuracy**	**0.828**	**0.632**	**0.991**	**0.914**	**1.000**	**0.989**	**0.837**	**0.633**
	**AUROC**	**0.899**	**0.681**	**0.997**	**0.759**	**1.000**	**0.912**	**0.903**	**0.677**
	**AUPRC**	**0.911**	**0.709**	**0.979**	**0.411**	**1.000**	**0.414**	**0.917**	**0.703**
RF	Accuracy	0.779	0.589	0.970	0.894	0.999	0.988	0.797	0.620
	AUROC	0.858	0.626	0.981	0.756	1.000	0.908	0.871	0.655
	AUPRC	0.872	0.657	0.928	0.344	0.999	0.335	0.885	0.682
XGBOOST	Accuracy	0.757	0.614	0.908	0.893	0.993	0.981	0.76	0.603
	AUROC	0.829	0.649	0.954	0.746	1.000	0.867	0.826	0.638
	AUPRC	0.848	0.675	0.777	0.366	0.999	0.273	0.842	0.668
LGBM	Accuracy	0.733	0.616	0.950	0.897	0.999	0.988	0.754	0.605
	AUROC	0.805	0.649	0.977	0.722	1.000	0.821	0.821	0.635
	AUPRC	0.826	0.677	0.873	0.331	1.000	0.297	0.841	0.664
ENRR	Accuracy	0.682	0.620	0.928	0.851	0.986	0.988	0.675	0.641
	AUROC	0.751	0.648	0.855	0.717	1.000	0.922	0.738	0.668
	AUPRC	0.773	0.676	0.608	0.379	0.994	0.363	0.762	0.697
ANN-MLP	Accuracy	0.646	0.595	0.931	0.770	0.999	0.982	0.723	0.624
	AUROC	0.736	0.674	0.827	0.622	1.000	0.930	0.798	0.683
	AUPRC	0.757	0.692	0.565	0.228	1.000	0.395	0.822	0.712

Mean model performance measures were reported in the *Training Set* and the *Test Set,* respectively.

*AATD-LD* = alpha-1 antitrypsin deficiency-associated liver disease, *AUPRC* = area under the precision-recall curve, *AUROC =* area under the receiver operating characteristic, *RF* = random forest, *XGBOOST* = extreme gradient boosting, *LGBM* = light gradient boosting, *ENRR* = elastic net regularized regression, *ANN-MLP* = artificial neural network multilayer perceptron.

Model performance measures from the stacking ensemble learning model are in bold.

**Table 7:** Overall predictive model performance measures of stacking ensemble and each base model used in the ML training in patients with any liver disease.

Model	Performance measure	All-cause mortality (N = 11,583)		Liver-related death (N = 11,583)		Liver transplant (N = 11,583)		All-cause mortality or liver transplant (N = 11,583)	
		Training	Test	Training	Test	Training	Test	Training	Test
**Stacking Ensemble Learning**	**Accuracy**	**0.806**	**0.756**	**0.991**	**0.913**	**0.999**	**0.989**	**0.815**	**0.755**
	**AUROC**	**0.852**	**0.770**	**0.999**	**0.835**	**1.000**	**0.859**	**0.863**	**0.777**
	**AUPRC**	**0.737**	**0.629**	**0.998**	**0.517**	**1.000**	**0.142**	**0.764**	**0.636**
RF	Accuracy	0.739	0.700	0.804	0.818	0.957	0.947	0.739	0.707
	AUROC	0.801	0.745	0.866	0.800	0.993	0.829	0.800	0.752
	AUPRC	0.676	0.577	0.784	0.391	0.906	0.114	0.688	0.601
XGBOOST	Accuracy	0.764	0.724	0.972	0.897	0.999	0.985	0.765	0.727
	AUROC	0.820	0.764	0.995	0.812	1.000	0.843	0.827	0.772
	AUPRC	0.685	0.606	0.991	0.461	1.000	0.112	0.710	0.631
LGBM	Accuracy	0.759	0.726	0.978	0.900	0.999	0.987	0.754	0.728
	AUROC	0.813	0.766	0.996	0.812	1.000	0.831	0.812	0.772
	AUPRC	0.678	0.609	0.994	0.465	1.000	0.105	0.691	0.632
ENRR	Accuracy	0.757	0.754	0.807	0.890	0.954	0.983	0.755	0.753
	AUROC	0.767	0.761	0.848	0.831	0.916	0.870	0.769	0.764
	AUPRC	0.616	0.608	0.766	0.507	0.406	0.143	0.636	0.630
ANN-MLP	Accuracy	0.767	0.746	0.886	0.878	0.988	0.979	0.769	0.740
	AUROC	0.785	0.762	0.945	0.824	0.994	0.735	0.790	0.776
	AUPRC	0.648	0.604	0.908	0.45	0.943	0.055	0.671	0.632

Mean model performance measures were reported in the *Training Set* and the *Test Set,* respectively.

*AUPRC* = area under the precision-recall curve, *AUROC* = area under the receiver operating characteristic, *RF* = random forest, *XGBOOST* = extreme gradient boosting, *LGBM* = light gradient boosting, *ENRR* = elastic net regularized regression, *ANN-MLP* = artificial neural network multilayer perceptron.

Model performance measures from the stacking ensemble learning model are in bold.

### Summary of feature importance

**Figure 6:**
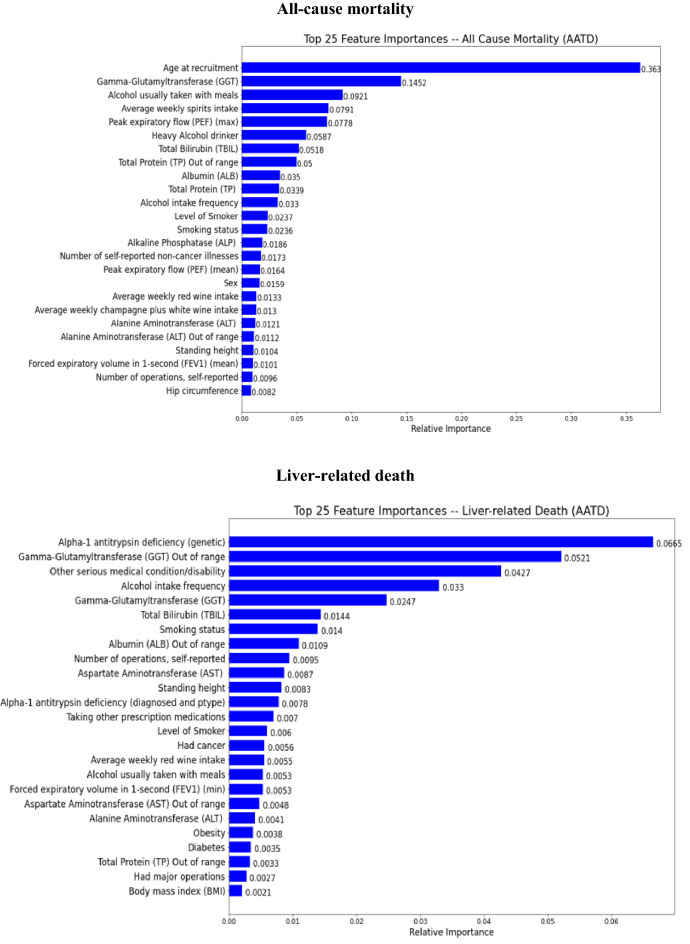

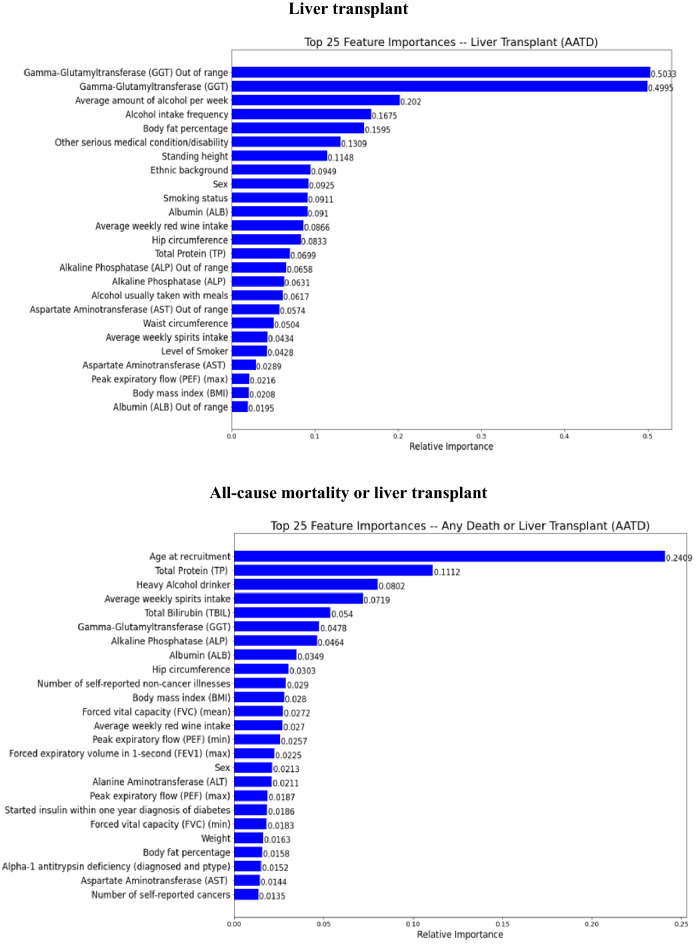
Feature importance in the final stacking ensemble learning model for patients AATD-LD (N = 455).

**Figure 7:**
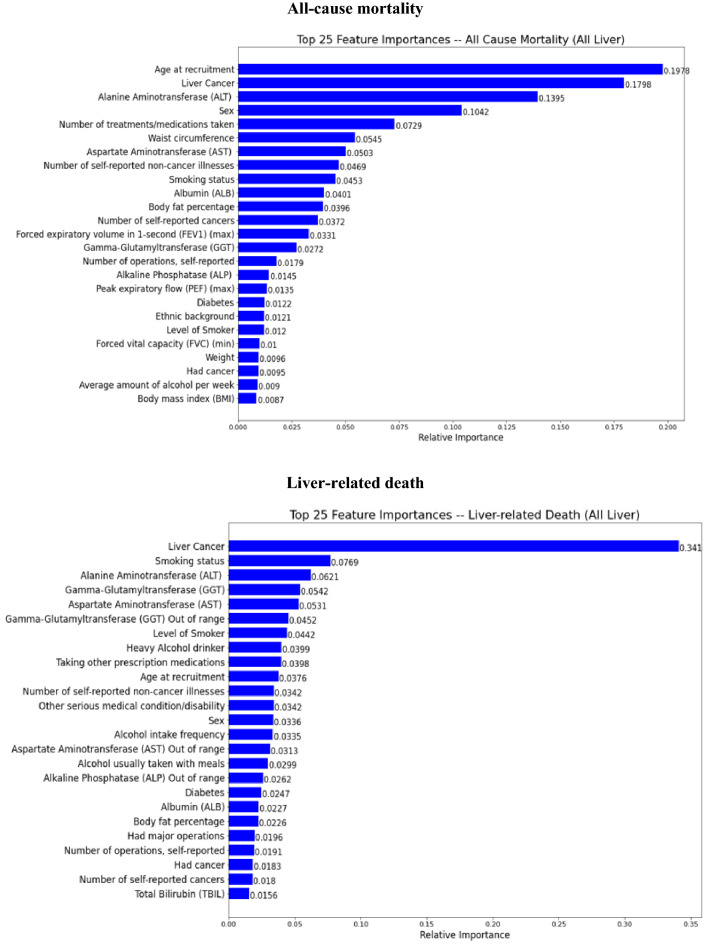

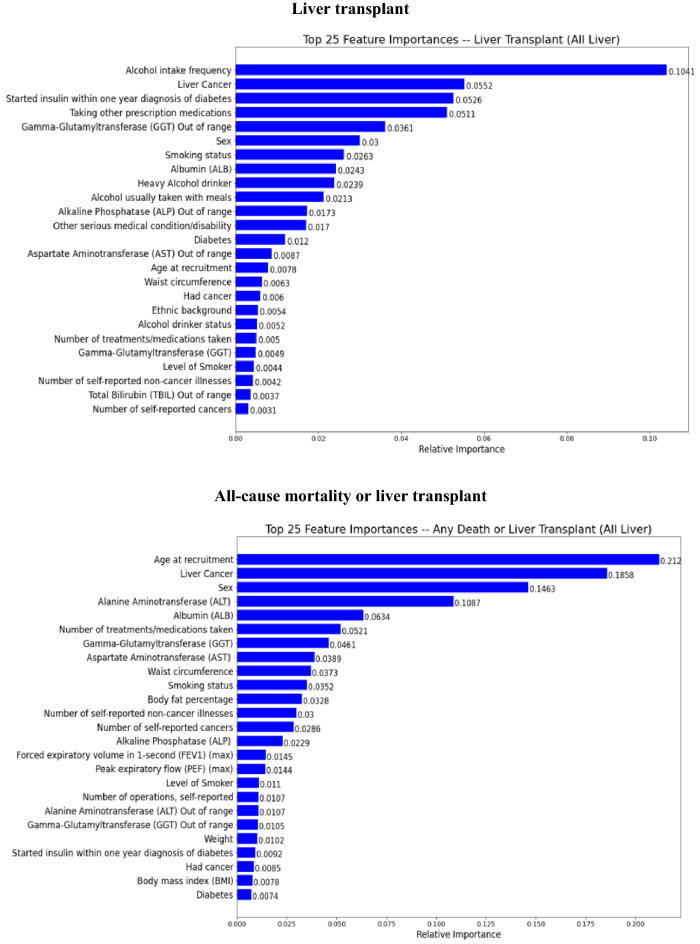
Feature importance in the final stacking ensemble learning model for patients with any liver disease (N = 11,583).

The top 25 important predictors ranked by their contributions to the outcome prediction in the final stacking ensemble learning predictive model among a total of 58 predictor variables were identified through a feature importance permutation method and presented in Figs. 6 and 7 in patients with AATD-LD and patients with any liver disease, respectively.

- *AATD-LD* Fig. 6 shows the top 25 important predictors for the stacking ensemble learning predictive model for patients with AATD-LD. The pattern of top-ranked predictors appeared similar, including baseline demographics (e.g., “age at recruitment”, “body fat percentage, “hip circumstance”, “standing height”), baseline disease characteristics (e.g., “number of self-reported non-cancer illness”, “number of self-reported operations”, “other serious medical conditions/disabilities”), liver function tests, lung function spirometry, alcohol intake and smoke status with the order slightly different for each clinical outcomes of disease progression. The highest-ranked predictors of all-cause mortality were age at recruitment, GGT, alcohol intake (e.g., “alcohol usually taken with meal”, “average weekly spirit intake”, “heavy alcohol drinker”, “alcohol intake frequency”), laboratory measurements (e.g., “total bilirubin”, “total protein out of range”, “total protein”, “albumin”,), lung function spirometry (e.g., max PEF), and smoking status (e.g., “level of smoker”, “smoking status”). The highest-ranked predictors of liver-related death were the genetic AAT deficiency (e.g., rs28929474 genotypes with Pi type of “ZZ”), GGT out of range, other serious medical conditions/disability, alcohol intake frequency, laboratory measurements (e.g., “GGT”, “total bilirubin”, “albumin out of range”, “AST”), smoking status, and number of self-reported operations. The highest-ranked predictors of liver transplant were GGT out of range, GGT, alcohol intake (e.g., “average amount of alcohol per week”, “alcohol intake frequency”), and baseline demographics and disease characteristics (e.g., “body fat percentage”, “other serious medication condition/disability”, “standing height”, “ethnic background”, “sex”). When combining all-cause mortality and liver transplant, the highest rank predictors were age at recruitment, total protein, alcohol intake (e.g., “heavy alcohol intake”, “average weekly spirits intake”), and laboratory measurements (e.g., “total bilirubin”, “GGT”, “ALP”, “albumin”), baseline demographics and disease characteristics (e.g., “hip circumstance”, “number of reported non-cancer illness”, “BMI”).
- *Any liver disease* Fig. 7 shows the top 25 important predictors for the stacking ensemble learning predictive model for patients with any liver disease. The prediction pattern was similar to patients with AATD-LD. It is interesting to note that the order of top-ranked predictors was slightly different from that in patients with AATD-LD. For example, the highest-ranked two predictors were age at recruitment and liver cancer for all-cause mortality, liver cancer and smoking status for liver-related death, alcohol intake frequency and liver cancer for liver transplant, and age at recruitment and liver cancer for all-cause mortality or liver transplant. It is worth noting that all three measures of alcohol intake (“alcohol intake frequency”, “heavy alcohol drinking”, and “alcohol usually taken with meals”) seem to play an important role in predicting liver transplant.

## Discussion

AATD-LD is a rare inherited genetic disease and is not well recognized. Since liver damage may progress slowly for decades before clinical presentation, it is crucial to predict the clinical outcomes of AATD-LD and define patients whose disease is more likely to progress in order to better understand the disease progression and promote timely medical intervention of AATD-LD in clinical practice. To date, there is little research in terms of disease progression of AATD-LD using the ML techniques. Hence, we developed a clinically meaningful and accurate predictive model using a novel stacking ensemble algorithm of disease progression of AATD-LD based on widely available clinical information. Our work proved the hypothesis that a combination of multiple ML models via meta-learning can produce a more powerful and robust ML predictive model, and it demonstrated the feasibility of applying such a novel ML technique to a large-scale complex and massive real-world database, the UK Biobank. Our work has provided a better understanding of the mechanism underlying the multivariate prediction of disease progression of AATD-LD. It has also enabled comparison of disease progression between AATD-LD and liver diseases in general.

### Advantages of stacking ensemble algorithm

The real challenge in the applied ML is to explore the space of possible ML models and identify a robust model with good prediction accuracy and reasonable interpretability^39^. The choice of the ML model depends on the specific data, e.g., data quantity, data dimension, data distribution, etc. We showed that the best ML performance was obtained from the proposed stacking ensemble predictive model by combining five base ML models via meta-learning. Our proposed stacking ensemble predictive model achieved an average accuracy of 63.2% and 75.6% for all-cause mortality, 91.4% and 91.3% for liver-related death, 98.9% and 98.9% for liver transplant, and 63.3% and 75.5% for all-cause mortality or liver transplant in prediction of AATD-LD and any liver disease, respectively, which surpassed each of the base ML models used in the ensemble. The stacking ensemble model worked particularly well in predicting liver-related death and liver transplant for both AATD-LD and any liver disease with a prediction accuracy greater than 90%.

It is noteworthy that no consistent trend in prediction performance was observed for the five base ML models in predicting four clinical outcomes of disease progression. For example, classification trees such as RF or GB appeared to work better for certain clinical outcomes such as liver-related death or liver transplant, whereas the regression method ENRR worked better for others. It shows that no single ML model is universally better or outperforms all others. It is worth noting that the stacking ensemble algorithm can combine classification trees with regression methods, and harness the benefits of these well-performing ML algorithms and enhance the prediction with superior performance than any single base ML model in the ensemble.

### Limitations of stacking ensemble algorithm

The common limitation of ML, lack of data or lack of good data, also applies to the stacking ensemble algorithm. An abundance of data is required to train and validate the ML model in order to produce useful results with good performance. The stacking ensemble algorithm also has its own limitations. For example, it may be time-consuming to build the model since each base ML model needs to be trained first. The stacking ensemble algorithm may also be harder to deploy and maintain, and less straightforward to interpret. Although the stacking ensemble model may not always be the best choice, the pros seem to outweigh the cons if properly used.

In addition, we would recommend the following in ML practice:

- apply an iterative imputation strategy to resolve model-fitting problems due to missing or incomplete information in the predictor variables;
- implement oversampling technique to overcome data imbalance challenges during model fitting process without overfitting; and
- utilize the nested k-fold cross-validation with repetitions to optimize the stability of the prediction results.

### Prediction of disease progression in AATD-LD

Our findings are generally consistent with the existing related work in AATD-LD based on conventional correlation or multivariate regression analysis but are more comprehensive, including four clinical outcomes of disease progression (i.e. all-cause mortality, liver-related death, liver transplant, all-cause mortality or liver transplant). Instead of relying on small-sized studies or older data, as did the existing research^7^, we used the complex and massive data in the UK Biobank which contains demographics, disease characteristics, medical history, lifestyle, physical activities, health outcome data, imaging and genetic data. The immense amount of data enabled us to build a ML predictive model for AATD-LD disease progression with clinically meaningful accuracy. The feature importance permutation method allowed us to rank the risk factors based on their contributions to the predictive model for a better understanding of disease progression of AATD-LD. All identified top-ranked predictors of each clinical outcome appeared clinically relevant.

1. *GGT* Different from some existing studies that showed mixed results for the serum tests as potential predictors^7,12,16–19^, our findings suggested liver function tests (e.g., GGT, total bilirubin) are among the top-ranked predictors for AATD-LD disease progression. In particular, it is interesting to note that GGT is usually dismissed as unimportant in predicting the course of chronic liver disease, because (1) it is more a sign of intrahepatic cholestasis and injury to the canalicular membrane or biliary epithelium than to hepatocytes; or even a sign of obstructive jaundice secondary to either intrahepatic or extrahepatic obstruction of bile ducts; and (2) it can easily be mildly perturbed by moderate alcohol intake, smoking, and multiple common medications. Our work showed that out-of-range GGT contributed more in predicting AATD-LD disease progression than other laboratory parameters that are considered more reflective of liver metabolism and transport (total bilirubin); liver synthetic function (albumin), hepatocyte injury (AST); or cholestasis (alkaline phosphatase). In a previous study of PiZZ AATD patients with lung and liver disease, GGT is also found in the lung and is related to static lung function, chronic bronchitis, sputum purulence, history of acute exacerbations, and smoking status in addition to alcohol consumption, cirrhosis and serum markers of liver disease^20^. GGT is independently correlated with airflow obstruction and is associated with chronic bronchitis and independently associated with mortality. This suggests that the importance of out-of-range GGT in AATD may originate from its dual source of origin in the two most affected and damaged organs in AATD, the liver and the lung. Our work may suggest the components of a clinical composite score that will help to predict disease progression to these clinical outcomes.
2. *Lung manifestations* Few studies have examined decline in lung function related to AATD-LD progression^7^. It is worth noting that lung manifestations of AATD (e.g., PEF) appeared among the top 10 important predictors of all-cause mortality but not liver-related mortality.
3. *Alcohol intake* Previous work showed inconsistent results in increased alcohol intake for AATD-LD progression^40,41^. Our findings suggested alcohol intake as a top-ranked predictor of AATD-LD progression. Of note there are multiple measurements of alcohol intake in the UK Biobank. Our work also showed the difference in term of contributions to the clinical outcomes among these alcohol intake measurements. For example, alcohol taken with meal, average weekly spirit intake, heavy alcohol drinker appeared to be among the top 10 ranked predictors for all-cause mortality, while alcohol intake frequency appeared important for liver-related death and liver transplant.

### Prediction of disease progression in AATD-LD versus any liver disease

The identified important predictor variables all appeared clinically relevant, although the predictor variables were slightly different for patients with AATD-LD and patients with any liver disease, which was not unexpected. For example, the genotype of PiZZ (e.g., rs28929474 genotypes with Pi type of “ZZ”) appeared to be the top one contributor to liver-related death and GGT to liver transplant in AATD-LD patients, while liver cancer was the top one contributor for liver-related death and alcohol intake for liver transplant in patients with any liver disease. Of note, only 20 AATD-LD patients had known genotype as PiZZ while the other patients’ genotype was either unknown or different from PiZZ. Thus, the disease progression for such patients is likely more dependent on something other than just a single allele Z mutation of the *SERPINA* gene (e.g., NASH, alcohol, other liver disease, other non-liver disease). This is supported by the pre-eminence of the PiZZ genotype as a predictor of disease progression in at least the 20 subjects who were known to carry that genotype. These results also suggest that in some AATD-LD patients the disease is driven by accumulation of Z-protein and inflammation, apoptosis and fibrosis in the liver, whereas in other patients the disease is likely primarily driven by something else but facilitated or exacerbated by accumulation of some Z-protein in the liver.

### Limitations and future research

Overall, our work demonstrates the feasibility of applying the ML technique to predict AATD-LD disease progression using the easily obtained demographic, baseline disease characteristics, lifestyle information and laboratory tests. Our work may lead to greater insights in clinical practice and assist clinicians in effectively identifying high-risk patients with AATD-LD, mitigating the burden of diagnosis and in managing the disease progression and treatment. It may also enable a data-driven strategy for biopharmaceutical companies to select clinical outcome endpoints and target patient populations in clinical research when developing a treatment for AATD-LD. However, there are a few limitations of this work. Firstly, given the data limitation of the UK Biobank, only the first 4 digits of ICD code were available to identify patients with AATD-LD, which might have affected the precision of AATD-LD patient selection. For example, E88.01 was the ICD10 code for AATD-LD, while only E88.0 was recorded in the UK Biobank. Secondly, there were very few AATD-LD patients with known genotype information in the UK Biobank, which limited our ability of further exploring the predictive pattern of disease progression in a subset of AATD-LD patients with PiZZ genotype. Lastly, one of the foci for liver disease research is to understand the patient disease progressive journey, in particular that of rapid disease progression. For future research, we will further explore the potential predictors of rapid disease progression of AATD-LD.

## Supplementary Information

Supplementary Information.

## Data Availability

The data underlying this article is a part of the UK Biobank dataset (application #26041) and is publicly available upon access request. The data, data processing, feature extraction, machine learning, and analysis code will be shared by the corresponding author upon reasonable request.

## References

[1] Nelson DR, Teckman J, Di Bisceglie AM, Brenner DA (2012). Diagnosis and management of patients with α1-antitrypsin (A1AT) deficiency. Clin. Gastroenterol. Hepatol..

[2] Kim M, Cai Q, Oh Y (2018). Therapeutic potential of alpha-1 antitrypsin in human disease. Ann. Pediatr. Endocrinol. Metab..

[3] Strnad P, McElvaney NG, Lomas DA (2020). Alpha_1_-antitrypsin deficiency. N. Engl. J. Med..

[4] Santos G, Turner AM (2020). Alpha-1 antitrypsin deficiency: An update on clinical aspects of diagnosis and management. Faculty Rev..

[5] de Serres FJ, Blanco I (2012). Prevalence of α1-antitrypsin deficiency alleles PI*S and PI*Z worldwide and effective screening for each of the five phenotypic classes PI*MS, PI*MZ, PI*SS, PI*SZ, and PI*ZZ: A comprehensive review. Ther. Adv. Respir. Dis..

[6] Elzouki AN, Eriksson S (1996). Risk of hepatobiliary disease in adults with severe alpha 1-antitrypsin deficiency (PiZZ): Is chronic viral hepatitis B or C an additional risk factor for cirrhosis and hepatocellular carcinoma?. Eur. J. Gastroenterol. Hepatol..

[7] Townsend SA (2018). Systematic review: The natural history of alpha-1 antitrypsin deficiency, and associated liver disease. Aliment. Pharmacol. Ther..

[8] American Thoracic Society, European Respiratory Society (2003). American Thoracic Society/European Respiratory Society statement: Standards for the diagnosis and management of individuals with alpha-1 antitrypsin deficiency. Am. J. Respir. Crit. Care Med..

[9] Wiegand J, Berg T (2013). The etiology, diagnosis and prevention of liver cirrhosis. Dtsch Arztebl. Int..

[10] Mitra S, De A, Chowdhury A (2020). Epidemiology of non-alcoholic and alcoholic fatty liver diseases. Transl. Gastroenterol. Hepatol..

[11] Hamesch K, Strnad P (2020). Non-invasive assessment and management of liver involvement in adults with alpha-1 antitrypsin deficiency. Chronic Obstr. Pulm. Dis..

[12] Tanash HA, Piitulainen E (2019). Liver disease in adults with severe alpha-1-antitrypsin deficiency. J. Gastroenterol..

[13] Pye A, Khan S, Whitehouse T, Turner AM (2021). Personalizing liver targeted treatments and transplantation for patients with alpha-1 antitrypsin deficiency. Exp. Rev. Precis. Med. Drug Dev..

[14] O’Brien ME (2015). The impact of smoke exposure on the clinical phenotype of alpha-1 antitrypsin deficiency in Ireland: Exploiting a national registry to understand a rare disease. COPD.

[15] Nakanishi T (2020). The undiagnosed disease burden associated with alpha-1 antitrypsin deficiency genotypes. Eur. Respir. J..

[16] Filipponi F (1994). Liver transplantation for end-stage liver disease associated with alpha-1-antitrypsin deficiency in children: Pretransplant natural history, timing and results of transplantation. J. Hepatol..

[17] Pferdmenges DC, Baumann U, Muller-Heine A, Framke T, Pfister E-D (2013). Prognostic marker for liver disease due to alpha1-antitrypsin deficiency. Klin. Padiatr..

[18] Pfister ED, Pferdmenges DC, Becker T, Rauschenfels S, Goldschmidt I, Baumann U (2011). Long-term outcome of alpha 1-antitrypsin deficiency related liver disease in children: A single-centre experience. JPGN.

[19] Volpert D, Molleston JP, Perlmutter DH (2000). Alpha1-antitrypsin deficiency-associated liver disease progresses slowly in some children. J. Pediatr. Gastroenterol. Nutr..

[20] Holme J, Dawkins PA, Stockley EK, Parr DG, Stockley RA (2010). Studies of gamma-glutamyl transferase in alpha-1-antitrypsin deficiency. COPD.

[21] Sidey-Gibbons JAM, Sidey-Gibbons CJ (2019). Machine learning in medicine: A practical introduction. BMC Med. Res. Methodol..

[22] Satapathy SK, Loganathan D (2022). Automated classification of multi-class sleep stages classification using polysomnography signals: A nine-layer 1D-convolution neural network approach. Multimed. Tools Appl..

[23] Satapathy SK, Loganathan D (2022). Automated classification of sleep stages using single-channel EEG: A machine learning-based method. IJIRR.

[24] Michielli N, Acharya UR, Molinari F (2019). Cascaded LSTM recurrent neural network for automated sleep stage classification using single-channel EEG signals. Comput. Biol. Med..

[25] Li Y, Peng C, Zhang Y, Zhang Y, Lo B (2022). Adversarial learning for semi-supervised pediatric sleep staging with single-EEG channel. Methods.

[26] Chen J, Manon G, Wong S, Kisfalvi K, Lirio RA (2022). Using supervised machine learning for treatment outcome prediction of vedolizumab in ulcerative colitis patients. J. Biopharm. Stat..

[27] Willetts M, Hollowell S, Aslett L, Holmes C, Doherty A (2018). Statistical machine learning of sleep and physical activity phenotypes from sensor data in 96,220 UK Biobank participants. Sci. Rep..

[28] Mohammed M, Mwambi H, Mboya IB, Elbashir MK, Omolo B (2021). A stacking ensemble deep learning approach to cancer type classification based on TCGA data. Sci. Rep..

[29] Miyoshi J (2021). Machine learning using clinical data at baseline predicts the efficacy of vedolizumab at week 22 in patients with ulcerative colitis. Sci. Rep..

[30] Oermann EK (2016). Using a machine learning approach to predict outcomes after radiosurgery for cerebral arteriovenous malformations. Sci. Rep..

[31] Wu CC (2019). Prediction of fatty liver disease using machine learning algorithms. Comput. Methods Progr. Biomed..

[32] Sudlow C (2015). UK biobank: An open access resource for identifying the causes of a wide range of complex diseases of middle and old age. PLoS Med..

[33] Elliott P, Peakman TC (2008). The UK Biobank sample handling and storage protocol for the collection, processing and archiving of human blood and urine. Int. J. Epidemiol..

[34] UK Biobank. *New Data & Enhancements to UK Biobank*. https://www.ukbiobank.ac.uk/enable-your-research/about-our-data (Accessed 12 July 2022).

[35] Chawla NV, Bowyer KW, Hall LO, Kegelmeyer WP (2002). SMOTE: Synthetic minority over-sampling technique. J. Artif. Intell. Res..

[36] Wolpert DH (1992). Stacked generalization. Neural Netw..

[37] Wang G, Hao J, Ma J, Jiang H (2011). A comparative assessment of ensemble learning for credit scoring. Expert Syst. Appl..

[38] Fisher A, Rudin C, Dominici F (2019). All models are wrong, but many are useful: Learning a variable's importance by studying an entire class of prediction models simultaneously. J. Mach. Learn. Res..

[39] Chen J, Kisfalvi K, Girard M, Wang S, Lirio RA (2022). Response to letter to editor. J. Bio. Stat..

[40] Triger DR, Millward-Sadler GH, Czaykowski AA, Trowell J, Wright R (1976). Alpha-1-antitrypsin deficiency and liver in adults. Q. J. Med..

[41] Bowlus CL (2005). Factors associated with advanced liver disease in adults with alpha1-antitrypsin deficiency. Clin. Gastroenterol. Hepatol..

