# Supplementary material for: A stacking ensemble machine learning model to predict alpha-1 antitrypsin deficiency-associated liver disease clinical outcomes based on UK Biobank data
Source: Sci Rep. 2022 Oct 11;12:17001. doi: 10.1038/s41598-022-21389-9 (PMC9554039; doi:10.1038/s41598-022-21389-9)
Supplement: Supplementary file 1 — Supplementary Information. [file 41598_2022_21389_MOESM1_ESM.docx]

**Appendix A. Stacking ensemble learning algorithm^1^.**

| **Algorithm: stacking ensemble learning** | | | |
| --- | --- | --- | --- |
|  | **Input:** Training dataset $D=\{\left( x_{1},y_{1} \right), \cdots,\left( x_{i},y_{i} \right),\cdots,{(x}_{n},y_{n})\}$;  Base-level ML algorithms $\mathcal{L}_{1},\cdots,\mathcal{L}_{5}$;  Meta-model algorithm $\mathcal{L}$. | | |
|  | **Output:** Classification result. | | |
| **1** | **For each model m**$\boldsymbol{=1, \cdots,5}$**do:** | | |
| **2** |  | Use the training dataset D to train a base-level ML learner $h_{m}=\mathcal{L}_{m}\left( D \right).$ | |
| **3** | **end** | | |
| **4** | Generate a new dataset $D^{'}$. | | |
| **5** | **For each individual i**$\boldsymbol{=1, \cdots,n}$**do:** | | |
| **6** |  | **For each model m**$\boldsymbol{=1, \cdots,5}$**do:** | |
| **7** |  |  | Use base-level ML learner $h_{m}$ to classify the training example $x_{i}$ and return the probability estimate $p_{im}= h_{m}(x_{i})$. |
| **8** |  | **end** | |
| **9** |  | Combine the probability estimates from 5 base-level ML learners $P_{i}=(p_{i1},p_{i2},\cdots,p_{i5})$. | |
| **10** |  | $D^{'}= D^{'}\cup\{(P_{i},y_{i})\}$. | |
| **11** | **end** | | |
| **12** | Use the new dataset $D^{'}$to train the meta-model learner $h^{'}\mathcal{=L(}D^{'})$. | | |
| **13** | Input a test example x and output the classification result $H\left( x \right)=h^{'}(h_{1}\left( x \right),h_{2}\left( x \right),\cdots,h_{5}\left( x \right))$. | | |

**Appendix B. Feature importance algorithm^2^.**

| **Algorithm: Permutation Importance** | | | |
| --- | --- | --- | --- |
|  | **Input:** Trained models $f_{1}, \cdots{,f}_{m}$, feature matrix $X$, target vector $y$, the **i**-th model performance measure AUROC$(y,f_{i}(X))$. | | |
|  | **Output:** Sorted feature importance. | | |
| **1** | **For each model** $\boldsymbol{i=1, \cdots,m}$**do:** | | |
| **2** |  | Estimate the original model performance measure $M_{i}^{orig}=AUROC(y,f_{i}(X))$ | |
| **3** |  | **For each feature j**$\boldsymbol{=1, \cdots,p}$**do:** | |
| **4** |  |  | Generate feature matrix $X^{perm}$by permuting feature **j** in the data $X$. |
| **5** |  |  | $M_{i}^{perm}=AUROC(y,f_{i}(X^{perm}))$ based on the predictions of the permuted data. |
| **6** |  |  | Calculate permutation importance ${FI}_{i}^{j}=M_{i}^{perm}- M_{i}^{orig}$. |
| **7** |  | **end** | |
| **8** | **end** | | |
| **9** | Final permutation importance for feature **j** across all models ${FI}^{j}=\sum_{i=1}^{m} {FI}_{i}^{j}$. | | |
| **10** | Sort features by descending FI. | | |

**Appendix C. Details of UK Biobank data domains and data processing in relation to Figure 2.**

This analysis was based on the UK Biobank data. The complete algorithms of patient identification and data processing were described as follows:

- **Patient grouping and information extraction**. Patients diagnosed with any liver disease were identified using International Classification of Diseases Tenth Revision code (ICD10) containing “K7, C22, E88.0, J43.8”. Patients diagnosed with AATD-LD were a subset of those with any liver disease and were identified using ICD10 code containing “E88.01, J43.8”, among whom 20 AATD-LD patients had their genotype as “PiZZ” collected via SNP rs28929474.
- **Preprocessing of predictor variables**. All potential predictor variables of AATD-LD and/or any liver disease were categorized into four predictor blocks to facilitate interpretation of prediction results: baseline demographics, baseline disease characteristics, lifestyle and others, and baseline laboratory measurements. There are multiple predictor variables within each predictor blocks. In order to prevent the modeling barriers from the overfitting or multicollinearity, redundant features were eliminated through feature selection methods. A predictor variable was included in the final model training if it was identified by at least 4 of the 7 feature selection methods (i.e., Pearson correlation, Chi-squared correlation, feature elimination recursive, Lasso, three tree-based models – random forest, extreme gradient boosting and light gradient boosting). In summary, a total of 58 predictor variables were identified and included in the final model training.
- **Clinical outcomes of interest.** The clinical outcomes of interest to assess the disease progression of AATD-LD and/or any liver disease included (1) all-cause mortality, taken from UK Biobank – death register; (2) liver-related death, which was a subset of all-cause mortality with liver disease diagnosis identified by ICD10 code; (3) liver transplant, taken from UK Biobank – summary of operations and identified by OPCS Classification of Interventions and Procedures code version 4 (OPCS4) containing “J01.1, Y99.2, Y99.3, Y99.5, Y99.6”; and (4) all-cause mortality or liver transplant, which was a combination of clinical outcomes (1), (2), and (3).

| **Category** | **Variables ^a^** | **UK Biobank**  **Data Domain(s)** | **Details of**  **Data Processing ^b, c^** |
| --- | --- | --- | --- |
| Patient Selection | Any liver disease | Summary Diagnoses | ICD10 codes (K7*, C22*, E88.0, J43.8) |
|  | AATD-LD | Summary Diagnoses | ICD10 codes (E88.01 and J43.8) |
| Clinical Outcomes | All-cause mortality | Death Register | Any death |
|  | Liver-related death | Death Register  Summary Diagnoses | Death with liver disease diagnosis code |
|  | Liver transplant | Operations | Operative procedure by OPSC4 code (J01.1, Y99.2, Y99.3, Y99.5, Y99.6) |
|  | All-cause mortality or liver transplant | Death Register  Operations | Any death or operative procedure by OPSC4 code |
| Predictor Block 1: Demographics | Age, Sex | Baseline Characteristics | NA |
|  | Ethnicity | Ethnicity | NA |
|  | Weight, Height, Waist circumstance, BMI | Body Size Measures | NA |
| Predictor Block 2:  Baseline disease characteristics | Other underlying diseases | Medical conditions  Medical information | NA |
| Predictor block 3:  Lifestyle and others​ | Lifestyle: smoking | Smoking | NA |
|  | Life style: alcohol intake | Alcohol | NA |
|  | Medical procedure | Operations | NA |
| Predictor block 4:  Baseline laboratory parameters | Laboratory parameters | Blood assay | NA |
|  | Lung function tests: FEV1, FVC, PEF | Spirometry | NA |
| ^a^ Detailed data fields used are available upon request.  ^b^ ICD10 = International Classification of Diseases Tenth Revision code, indicates laterality, stage of care, specific diagnosis, and specific anatomy.  ^c^ OPCS4 = OPCS Classification of Interventions and Procedures code version 4, is the [procedural classification](https://en.wikipedia.org/wiki/Procedure_code) for**operations, procedures and interventions performed during in-patient stays, day case surgery and some out-patient treatments in** [National Health Service](https://en.wikipedia.org/wiki/National_Health_Service) (NHS) hospitals of [NHS England](https://en.wikipedia.org/wiki/National_Health_Service_(England)), [NHS Scotland](https://en.wikipedia.org/wiki/NHS_Scotland), [NHS Wales](https://en.wikipedia.org/wiki/NHS_Wales) and [Health and Social Care in Northern Ireland](https://en.wikipedia.org/wiki/Health_and_Social_Care_in_Northern_Ireland). | | | |

**References**

1. Wolpert, D. H. Stacked generalization. *Neural Netw.* **5**, 241–259 (1992). <https://doi.org/10.1016/S0893-6080(05)80023-1>
2. Fisher, A., Rudin, C. & Dominici, F. All models are wrong, but many are useful: learning a variable's importance by studying an entire class of prediction models simultaneously. *J. Mach. Learn. Res.* **20**, 177 (2019). https://doi.org/10.48550/arXiv.1801.01489
